# Supplementary material for: Mutations in TIMM50 compromise cell survival in OxPhos‐dependent metabolic conditions
Source: EMBO Mol Med. 2018 Sep 6;10(10):e8698. doi: 10.15252/emmm.201708698 (PMC6180300; doi:10.15252/emmm.201708698)
Supplement: Supplementary file 1 — Appendix [file EMMM-10-e8698-s001.pdf]

Appendix

Inventory of Appendix

Case report and clinical discussion

Appendix Figure S1

Appendix Figure S2

Appendix Figure S3

Appendix Figure S4

Appendix Figure S5

Appendix Figure S6

Appendix Figure S7

Appendix Figure S8

Appendix Figure S9

Appendix Figure S10

Appendix Figure S11

Appendix Table 1

Case report and clinical discussion

The patient (born in 2010) was the first daughter of non-consanguineous Italian parents. The pregnancy was uneventful and the child was born at term without complication. Her birth weight was 2470 g (<3° percentile), length was 49 cm (50° percentile) and her head circumference was 33 cm (25-50° percentile). Slight hypotonia, poor sucking and failure to thrive were present from birth.

At 2 months of age, she presented with infantile spasms associated with hypsarrhythmia at the EEG. These episodes lasted 10-20 minutes with increasing frequency, from 2-3 times per week to 4-5 times per day, during one month. Psychomotor regression included loss of eye contact.

At the age of 6 months, the patient was transferred to the Unit for metabolic investigations due to persistent lactic acidosis. Lactate was elevated both in plasma (5.6 mmol/L, n.v. <1.8) and cerebrospinal fluid (3,6 mmol/L), plasma amino acids showed high alanine (620 umol/L; n.v. 143-439). Urinary organic acid showed increased lactate and Krebs cycle metabolites (succinate and malate), but 3-methylglutaconic aciduria was never detected.

A muscle biopsy, performed at the age of 4 months, showed reduced activity of MRC complex II and succinate dehydrogenase.

Electroretinogram (ERG) and visual evoked potential (VEP) were abnormal.

Brain MRI (Figure S1) showed moderate brain atrophy and symmetric lesions of thalami (white arrowheads), cerebral peduncles (red arrowheads), and ponto-mesencephalic tegmentum (white arrows). The MRI spectroscopy of the brain cortex disclosed a lactate peak.

Coenzyme Q10, Vitamins A and E, lipoic acid and riboflavin were added to the antiepileptic treatment (vigabatrim) without benefit in decreasing plasma lactate.

At 17 months of life the patient had her first respiratory arrest, probably due to an ab ingestis pneumonia with dramatic worsening of her neurological status. The patient died at the age of 32 months for cardiorespiratory arrest.

TIMM50 mutations have been very recently described in two couples of siblings with mitochondrial epileptic encephalopathy and 3-methylglutaconic aciduria (Shahrour et al., 2017). The clinical phenotype of our patient, showing early-onset hypotonia, psychomotor regression/stagnation, and severe epilepsy, with Leigh-like MRI features resembled the reported patients. The presence of lactic acidosis was a common feature of all the *TIMM50* mutant subjects but the 3-methylglutaconic aciduria, reported as a hallmark of the *TIMM50* patients by Sharour et al., was absent in our patient. Concerning the MRC activities, Shahrour et al. reported partial complex V deficiency in muscle of one patient and normal mitochondrial respiratory chain activities in a second one. The measurement of the complex V activity (as oligomycin sensitive ATPase) is tricky, and the assay is not reliable in frozen muscle (Diodato et al., 2015). Thus, we did not assess this complex in patient's muscle biopsy. Taken into account all these considerations, we suggest to be cautious about the link between complex V deficiency and *TIMM50* mutations. Instead, the biochemical analysis in muscle biopsy from our patient showed a clear complex II deficiency (both SQR and SDH activities; 33% and 44% of mean value, respectively). In addition, immunoblot analysis of patient cells showed reduced levels of subunits of complex II and complex IV and of one subunit of complex I, leading to decrease in respiration. Overall, the clinical presentation and the progression of our patient was more severe, leading to a fatal outcome at 2 years while the other *TIMM50* mutant subjects were still alive at 10-15 years of age. The fact that our patient was compound heterozygous for a nonsense and a missense mutation, and that she showed a profound reduction in TIMM50 amount, whereas the other *TIMM50* mutant patients were homozygous for missense mutations (the levels of TIMM50 protein was not assessed), may explain the different severity. In

addition, the mutation in our patient maps in the transmembrane domain while mutations in the other patients are localized in the domain protruding into the intermembrane space which forms the presequence binding domain (PBD), either in the linker region (p.R217W) or in the FCP1-like domain (p.T252M).

Diodato, D., Invernizzi, F., Lamantea, E., Fagiolari, G., Parini, R., Menni, F., Parenti, G., Bollani, L., Pasquini, E., Donati, M.A., et al. (2015). Common and Novel TMEM70 Mutations in a Cohort of Italian Patients with Mitochondrial Encephalocardiomyopathy. JIMD Rep *15*, 71-78.

Shahrour, M.A., Staretz-Chacham, O., Dayan, D., Stephen, J., Weech, A., Damseh, N., Pri Chen, H., Edvardson, S., Mazaheri, S., Saada, A., et al. (2017). Mitochondrial epileptic encephalopathy, 3-methylglutaconic aciduria and variable complex V deficiency associated with TIMM50 mutations. Clin Genet *91*, 690-696.

Informed consent was obtained from all subjects and the experiments conformed to the principles set out in the WMA Declaration of Helsinki and the Department of Health and Human Services Belmont Report. The Ethical Committee of the Fondazione IRCCS Istituto Neurologico 'Carlo Besta', Milan (Italy) approved the present study.

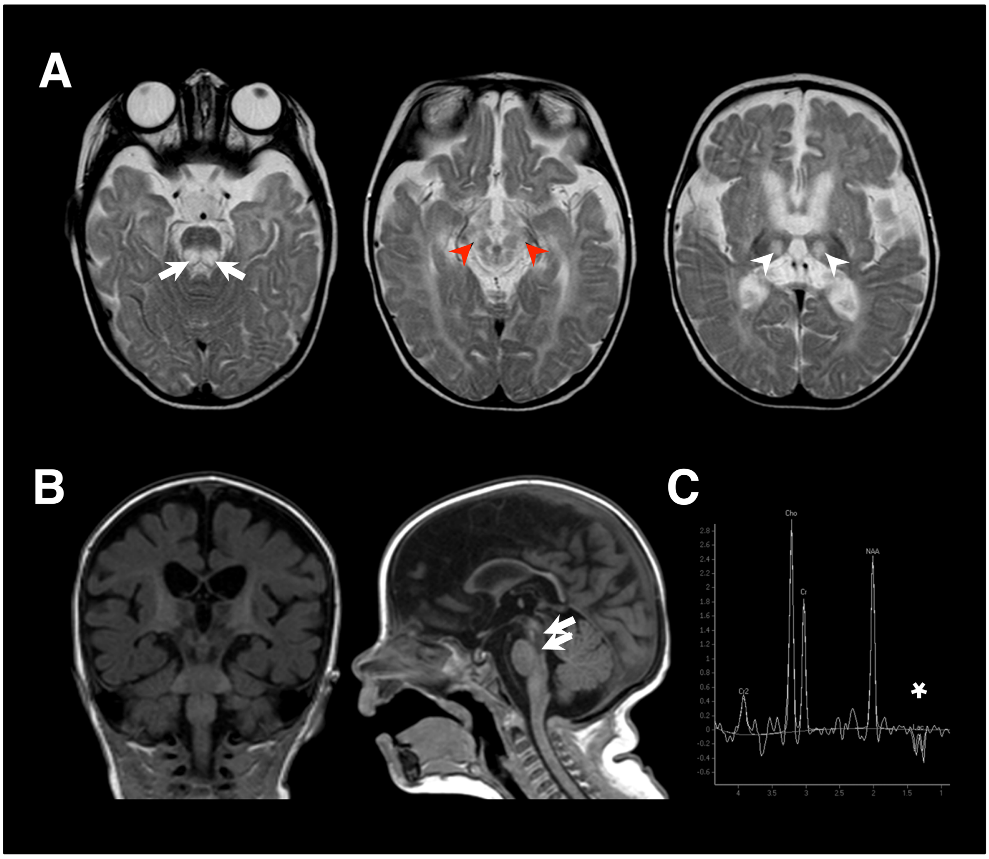

Appendix Figure S1. Patient brain MRI

A. axial T2-weighted

B. coronal and sagittal T1-weighted.

C. MRI spectroscopy disclosing a peak of lactate (*).

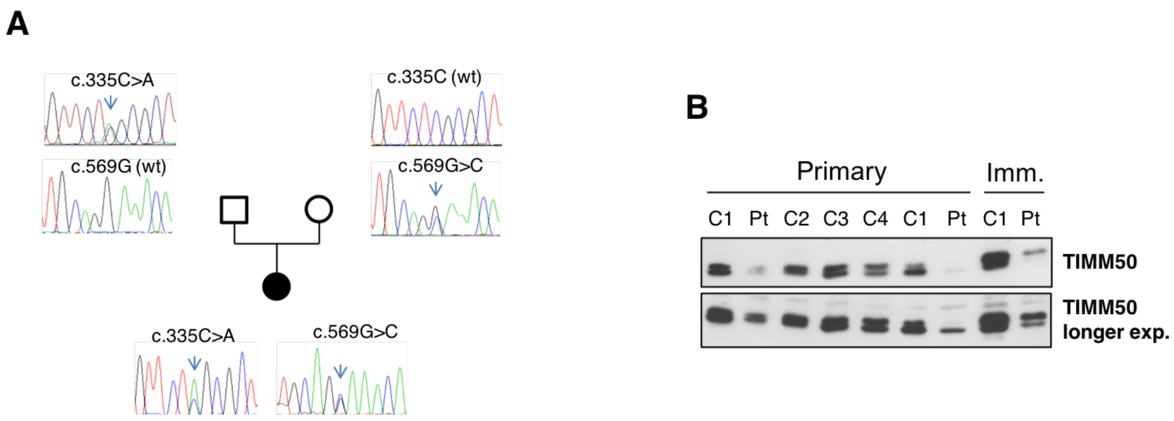

Appendix Figure S2. Sanger sequencing and TIMM50 levels

A. Sanger sequence verification of the *TIMM50* mutations found by exome sequence.

B. Longer exposure of endogenous TIMM50S levels in control and TIMM50 mutant fibroblasts shown in Figure 1C

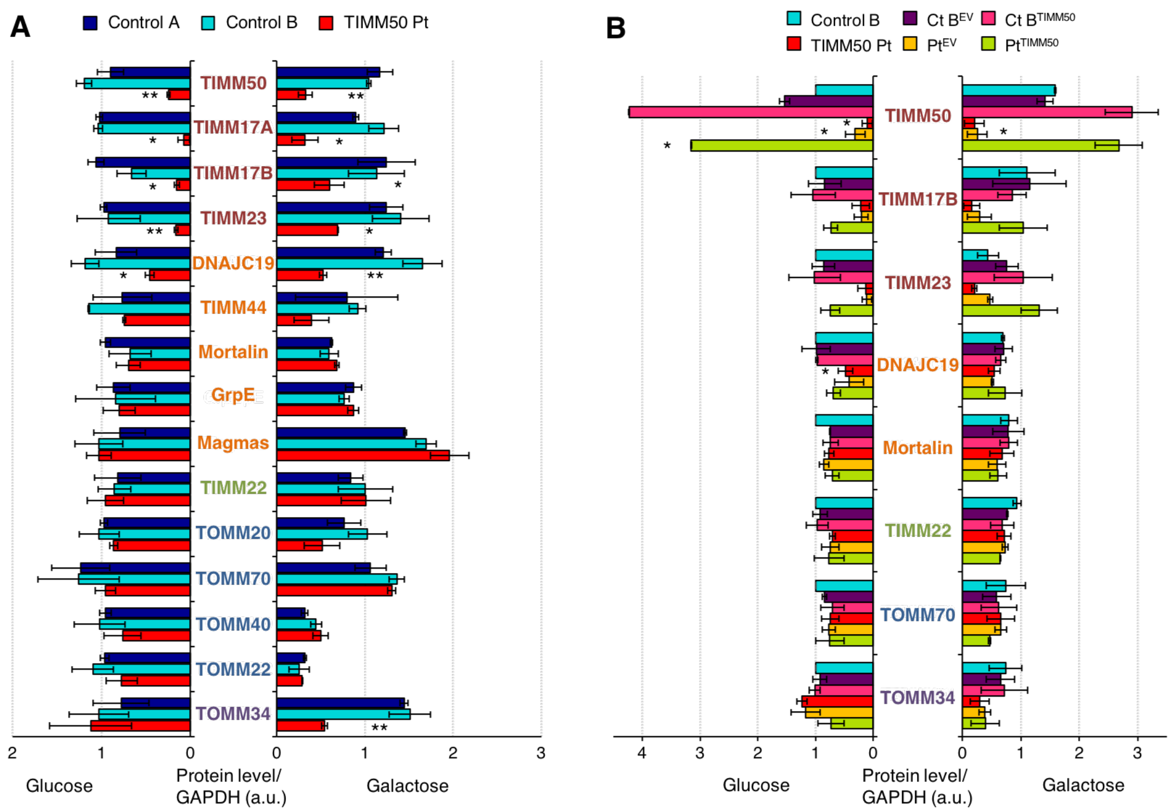

Appendix Figure S3. Quantification of proteins of the import machinery

A. Quantification of the Western-blots shown in Fig 2A for Control A and B and TIMM50 mutant fibroblasts using GAPDH for normalization. Data are shown as mean ± SD, n = 2 biological replicates; $^{*}p < 0.05$, $^{**}p < 0.01$ grouping both controls together.

B. Quantification of the Western-blots shown in Fig 2B for control and *TIMM50* mutant fibroblasts, complemented with the empty vector (EV) or TIMM50 using GAPDH for normalization. Data are shown as mean ± SD, n = 2 biological replicates; $^{*}p < 0.05$.

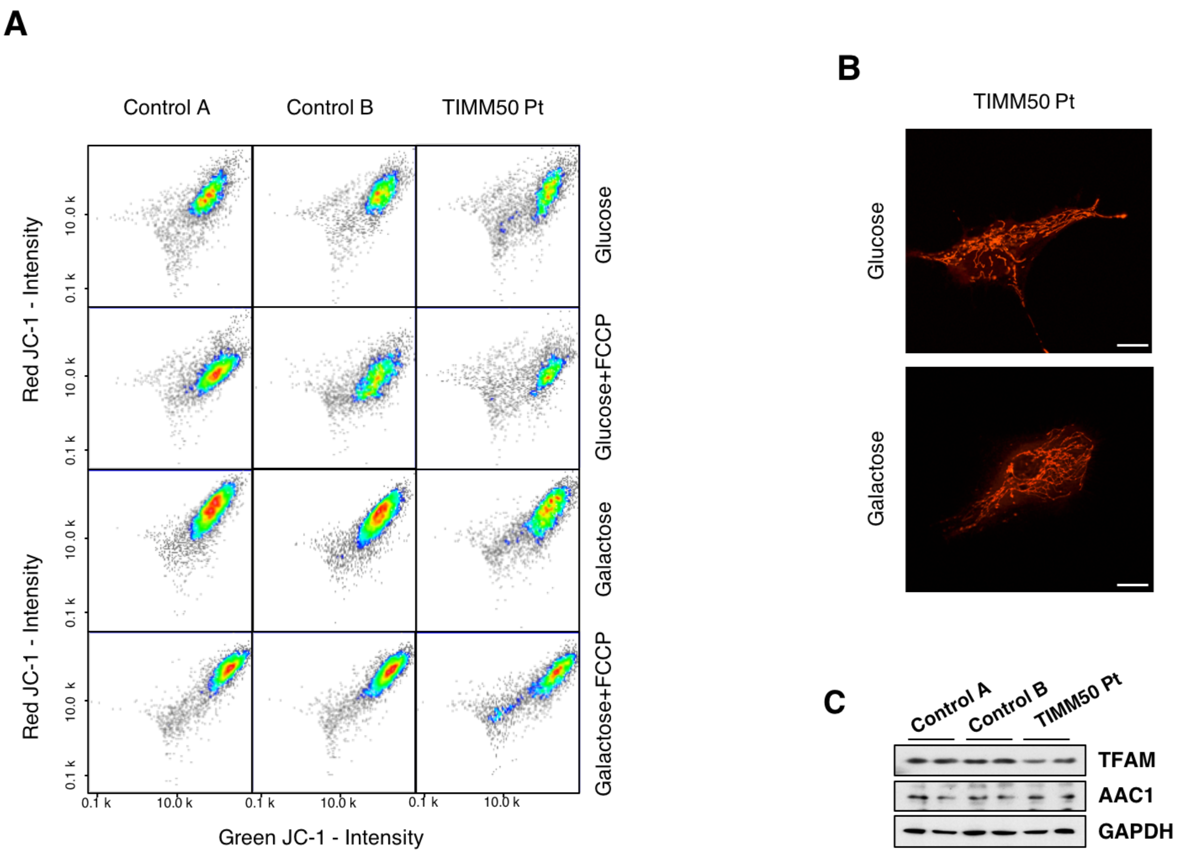

Appendix Figure S4. Mitochondrial membrane potential and protein levels

A. Plots of green JC-1 against red JC-2 intensity in control and *TIMM50* mutant fibroblasts grown in glucose or galactose. Cells were left untreated or treated with 1μM FCCP for 5 min at 37°C.

B. Digitally enhanced image of *TIMM50* mutant fibroblasts grown in glucose or galactose after treatment with TMRM, as in Fig 2D. Scale bar corresponds to 10 μm.

C. Steady-state levels of TFAM and AAC1 compared to GAPDH in control and *TIMM50* mutant fibroblasts grown in glucose.

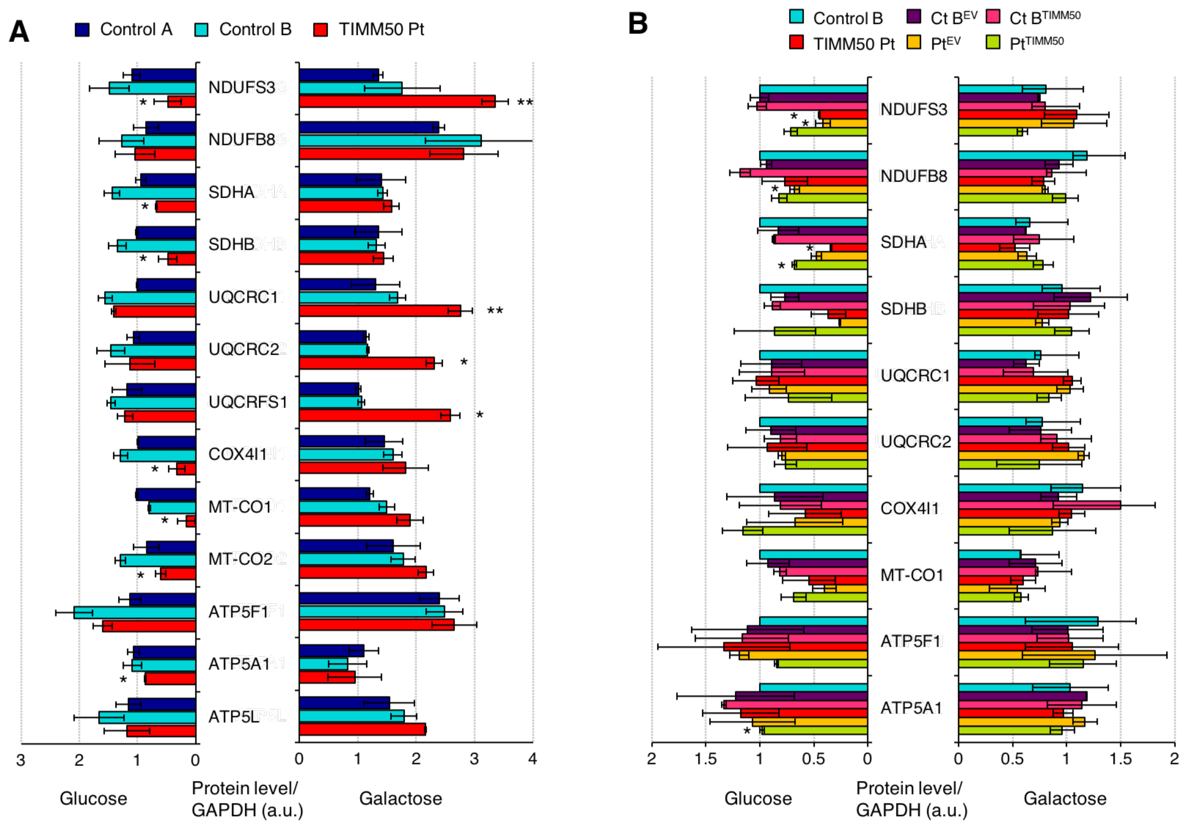

Appendix Figure S5. OxPhox protein levels

A. Quantification of the Western-blots shown in Fig 4A for control and *TIMM50* mutant fibroblasts using GAPDH for normalization. Data are shown as mean ± SD, n = 2 biological replicates; $^{*}p < 0.05$, $^{**}p < 0.01$ grouping both controls together.

B. Quantification of the Western-blots shown in Fig 4B for control and *TIMM50* mutant fibroblasts, complemented with the empty vector (EV) or TIMM50 using GAPDH for normalization. Data are shown as mean ± SD, n = 2 biological replicates; $^{*}p < 0.05$.

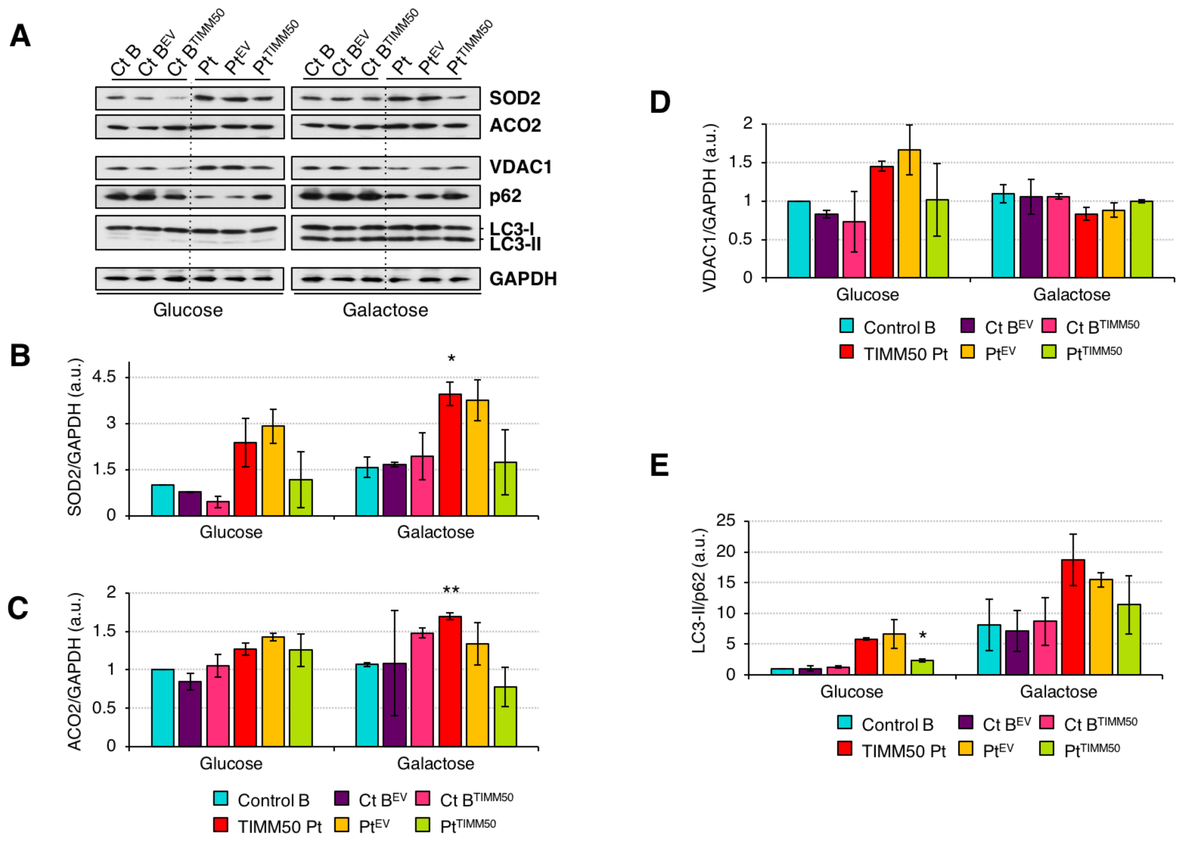

Appendix Figure S6. Steady state levels of ROS and mitophagy related proteins

A. Steady state levels of ROS related proteins SOD2 and ACO2 and mitophagy related proteins VDAC1, p62 and LC3-II in control and TIMM50 mutant fibroblasts, transduced with the empty vector (EV) or with wild-type TIMM50 growing in either glucose or galactose were assessed by Western blot using GAPDH as internal control.

B-E. Quantification of the steady state levels of proteins in control and *TIMM50* mutant fibroblasts, transduced with the empty vector (EV) or with wild-type TIMM50 growing in either glucose or galactose. Data are shown as mean ± SD, n = 2 biological replicates; $^{*}p < 0.05$; $^{**}p < 0.01$.

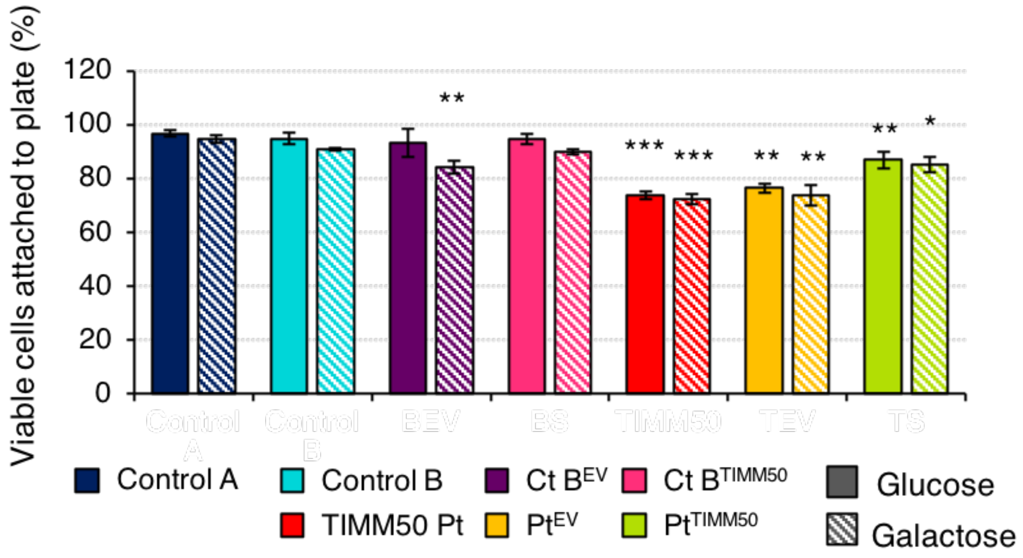

Appendix Figure S7. ***Cell viability***

Number of viable cells as measured by Trypan blue in control and *TIMM50* mutant fibroblasts, transduced with the empty vector (EV) or with wild-type TIMM50 grown in glucose or galactose. Data are shown as mean ± SD, n = 8; $^{*}p < 0.05$, $^{**}p < 0.01$, $^{***}p < 0.001$

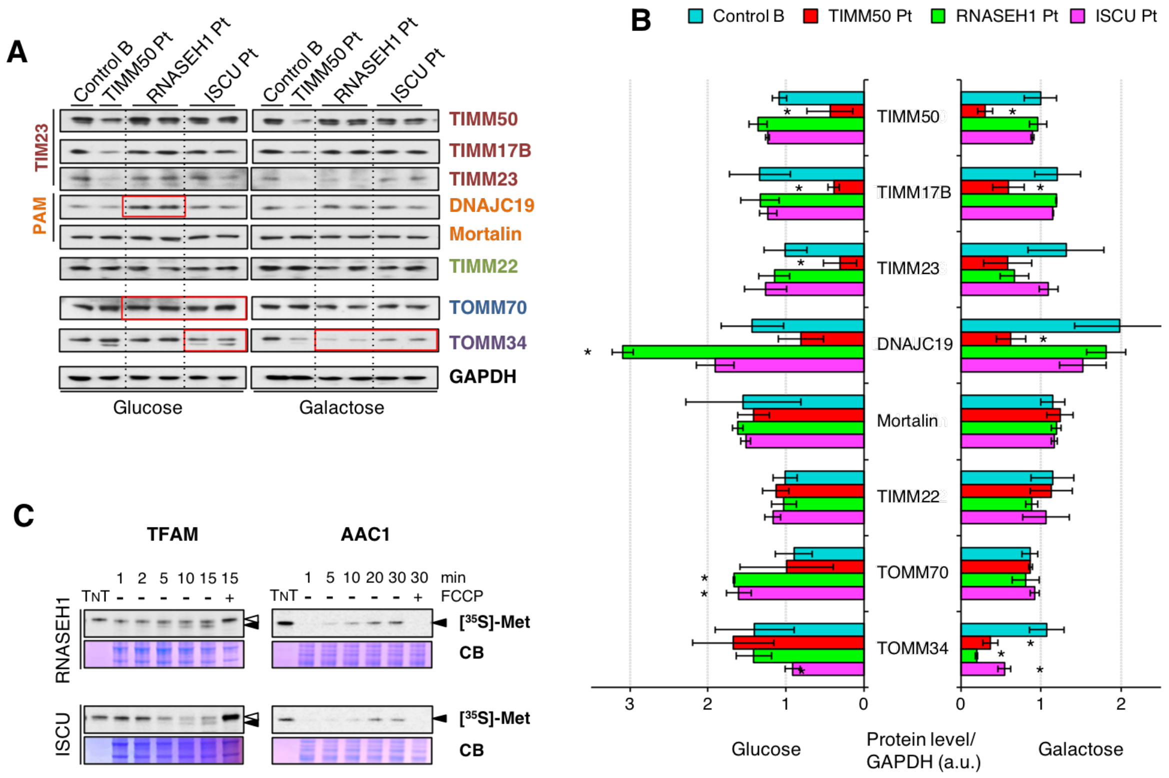

Appendix Figure S8. Import protein levels and import activity

A. Western-blot of whole cell extracts for different components of the protein import machinery in control and *TIMM50, RNASEH1* and *ISCU* mutant fibroblasts grown in either glucose or galactose containing medium.

B. Quantification of the Western-blots shown in A for control and mutant fibroblasts using GAPDH for normalization. Data are shown as mean ± SD, n = 2 biological replicates; $^{*}p < 0.05$.

C. *In organello* import of the precursors of TFAM and AAC1 into isolated mitochondria from *RNASEH1* and *ISCU* mutant fibroblasts in the absence or presence of the uncoupler FCCP. The radiolabeled precursors were incubated with isolated mitochondria for the indicated times followed by trypsin treatment only in the case of AAC1. Coomassie Blue (CB) staining was used as loading control and the radiolabeled protein (TNT) as reference. The position of the precursor (empty arrowheads) and the mature protein (filled arrowhead) are also shown.

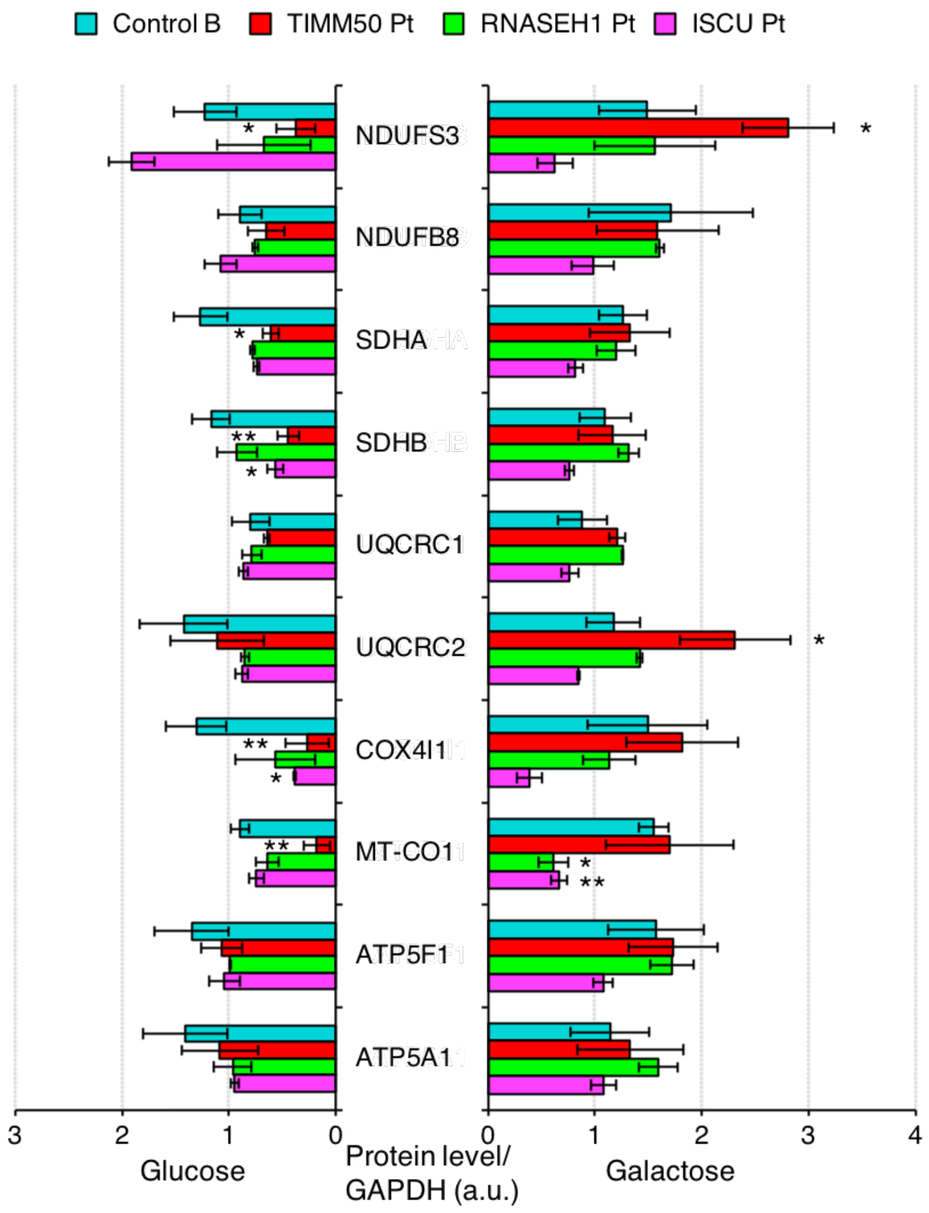

Appendix Figure S9. OxPhox protein levels

Quantification of the Western-blots shown in Fig 7C for control and *TIMM50, RNASEH1* and *ISCU* mutant fibroblasts using GAPDH for normalization. Data are shown as mean ± SD, n = 2 biological replicates; $^{*}p < 0.05$, $^{**}p < 0.01$

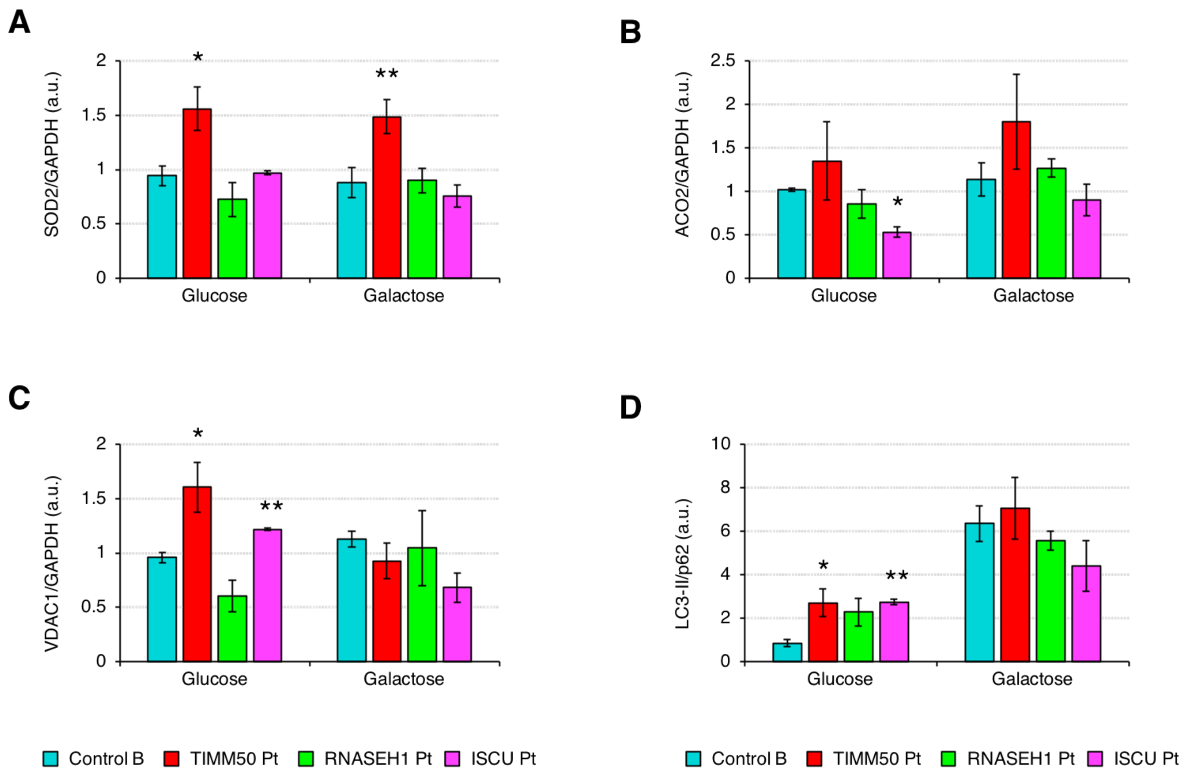

Appendix Figure S10. Steady state levels of ROS and mitophagy related proteins

A-D. Quantification of the steady state levels of proteins in control and *TIMM50, RNASEH1* and *ISCU* mutant fibroblasts growing in either glucose or galactose as shown in Fig 7E. Data are shown as mean ± SD, n = 2 biological replicates; $^{*}p < 0.05$, $^{**}p < 0.01$.

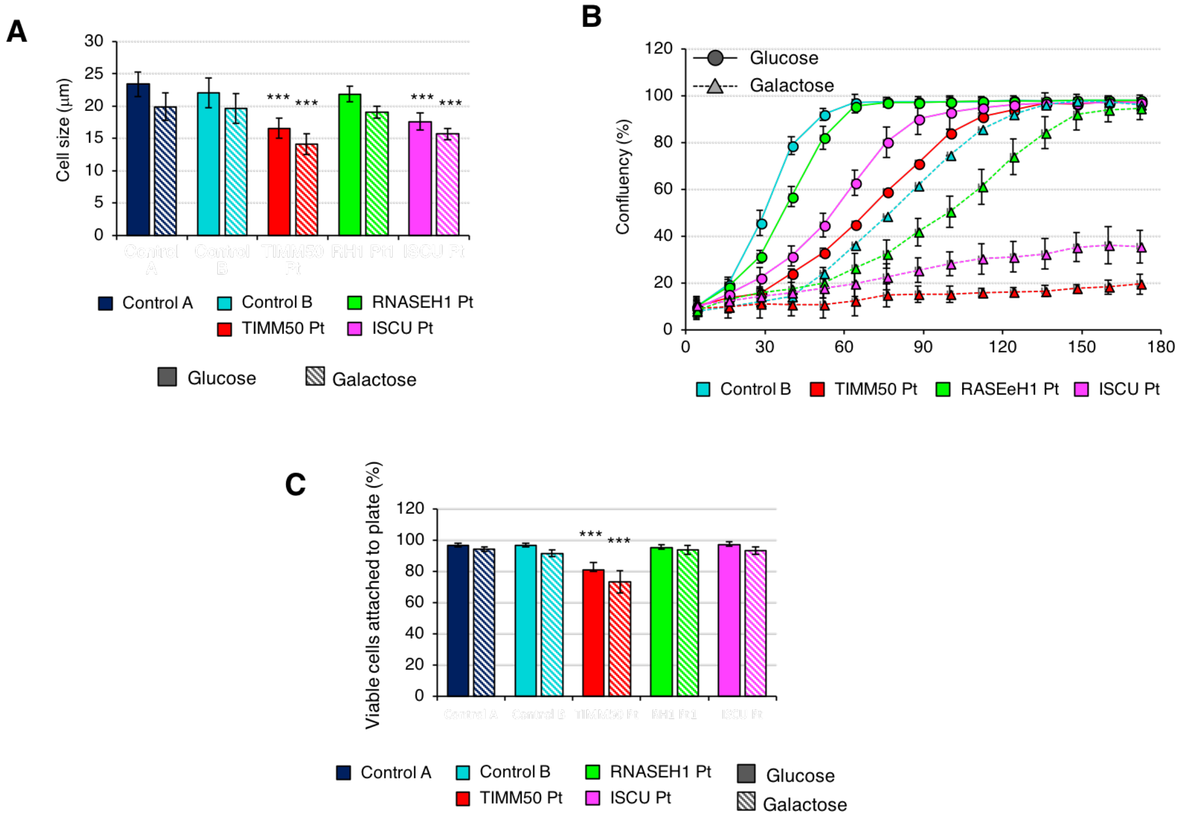

Appendix Figure S11. Cell size, growth rate and cell viability

A. Cell size in control and *TIMM50, RNASEH1* and *ISCU* mutant fibroblasts grown in glucose or galactose. Data are shown as mean ± SD, n = 5 biological replicates; $^{***}p < 0.001$

B. Growth curves of control and *TIMM50, RNASEH1* and *ISCU* mutant fibroblasts grown in glucose or galactose. Cell growth was monitored continuously by the Incucyte live cell imager (Essen Bioscience). One of the three independent experiments carried out is presented. Data are shown as mean of three technical replicates ± SD.

C. Number of viable cells as measure by Trypan blue in control and *TIMM50, RNASEH1* and *ISCU* mutant fibroblasts grown in glucose or galactose. Data are shown as mean ± SD, n = 5; $^{***}p < 0.001$

Antibody	Company	Catalog number	Working dilution
TIMM50	Proteintech	22229-1-AP	1:5,000
TIMM17A	Abcam	ab192246	1:1,000
TIMM17B	Abcam	ab122034	1:2,000
TIMM23	Sigma	101-113	1:1,000
DANJC19	Proteintech	12096-1-AP	1:1,000
TIMM44	Abcam	ab201453	1:2,000
Mortalin	Proteintech	14887-1-AP	1:10,000
GrpE	Proteintech	12720-1-AP	1:1,000
Magmas	Proteintech	15321-1-AP	1:1,000
TIMM22	Abcam	ab167423	1:1,000
TOMM20	Santa Cruz	sc-11415	1:5,000
TOMM70	Abcam	ab89624	1:1,000
TOMM40	Proteintech	18409-1-AP	1:2,000
TOMM22	Novus	NBP1-80671	1:1,000
TOMM34	Abcam	ab103585	1:1,000
GAPDH	Abcam	ab8245	1:15,000
NDUFS3	Abcam	ab110246	1:1,000
NDUFB8	Abcam	ab110242	1:1,000
SDHA	Abcam	ab14715	1:5,000
SDHB	Abcam	ab14714	1:5,000
UQCRC1	Abcam	ab96333	1:5,000
UQCRC2	Abcam	ab14745	1:5,000
UQCRFS1	Abcam	ab14746	1:5,000
COX4I1	Abcam	ab14744	1:2,000
MT-CO1	Abcam	ab14705	1:2,000
MT-CO2	Abcam	ab91317	1:2,000
ATP5F1	Abcam	ab84625	1:5,000
ATP5A1	Abcam	ab110273	1:10,000
SOD2	Abcam	ab16956	1:2,000
ACO2	Abcam	ab110321	1:2,000
VDAC1	Abcam	ab14734	1:2,000
p62	Sigma	P0067	1:10,000
LC3	Novus	NB100-2220	1:2,000

Appendix Table 1. List of antibodies used in the present study
