# Supplementary figures and images for: Mutations in TIMM50 compromise cell survival in OxPhos‐dependent metabolic conditions
Source: EMBO Mol Med. 2018 Sep 6;10(10):e8698. doi: 10.15252/emmm.201708698 (PMC6180300; doi:10.15252/emmm.201708698)

1C

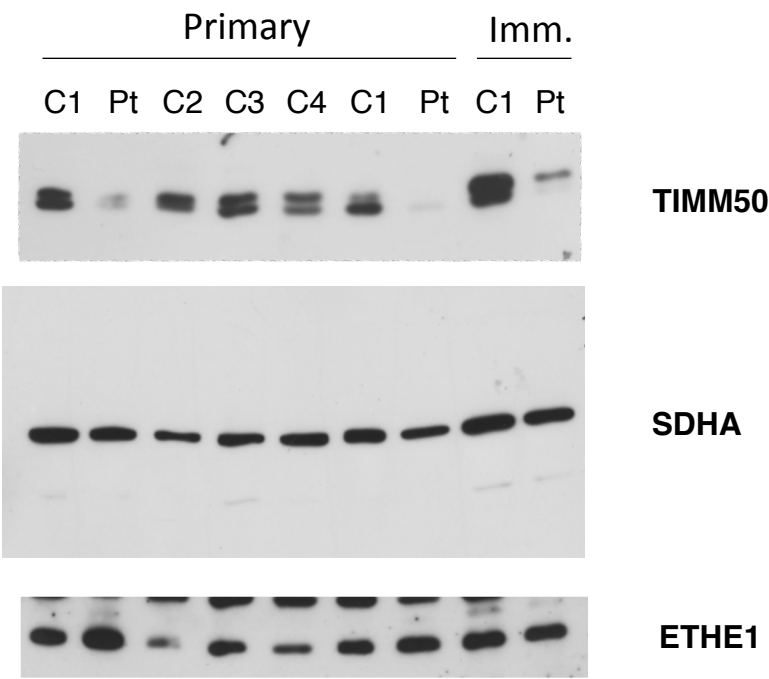

Supplement: Supplementary file 4 — Source Data for Figure 1 [file EMMM-10-e8698-s003.pdf]

2A

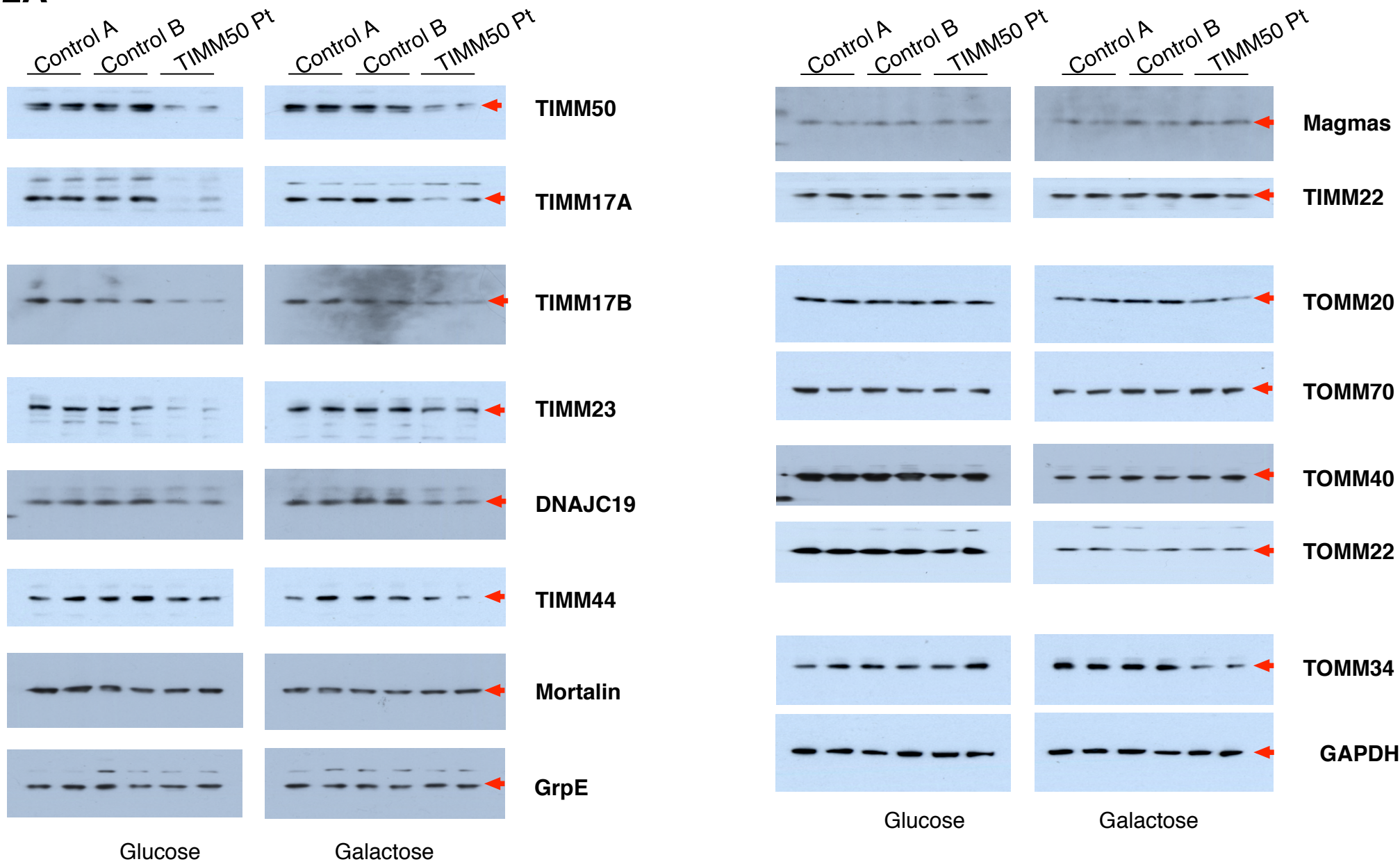

2B

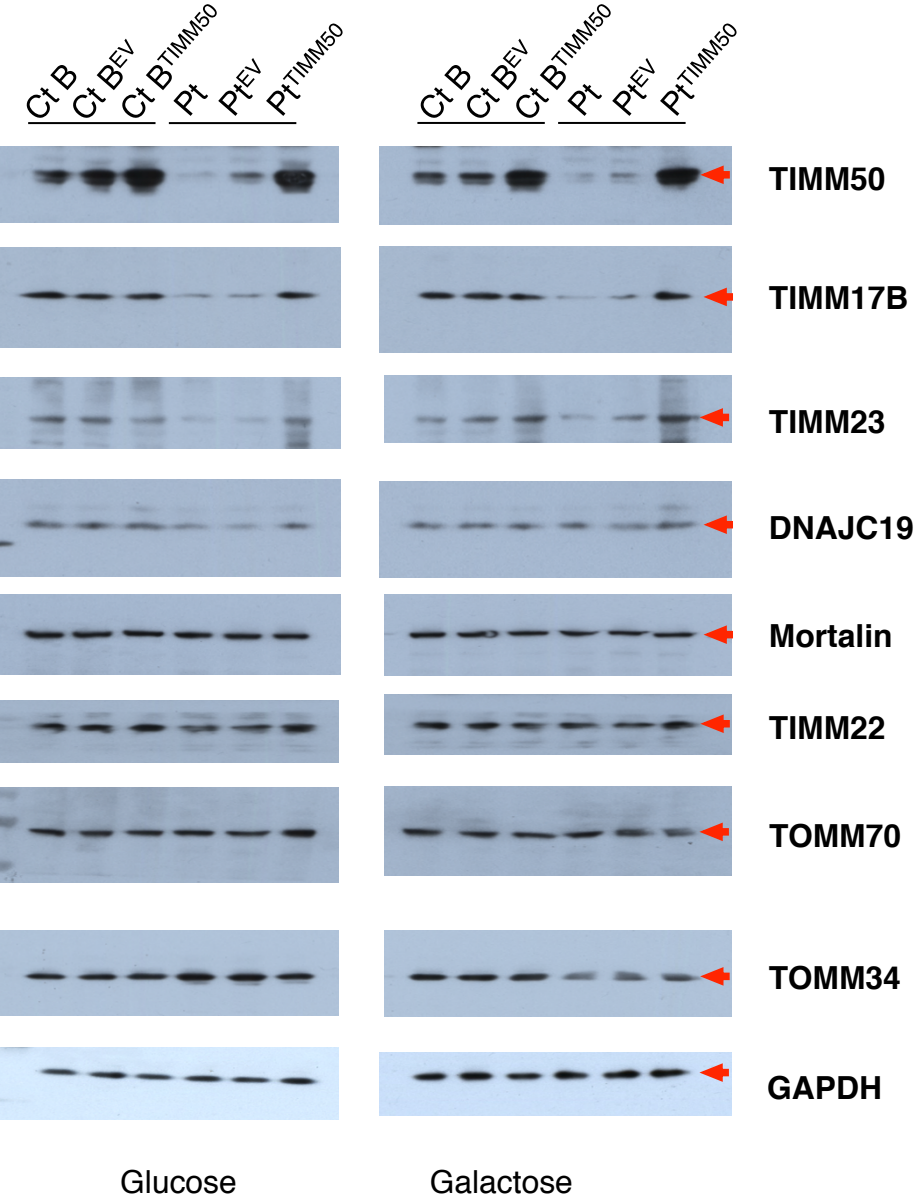

Supplement: Supplementary file 5 — Source Data for Figure 2 [file EMMM-10-e8698-s004.pdf]

3A

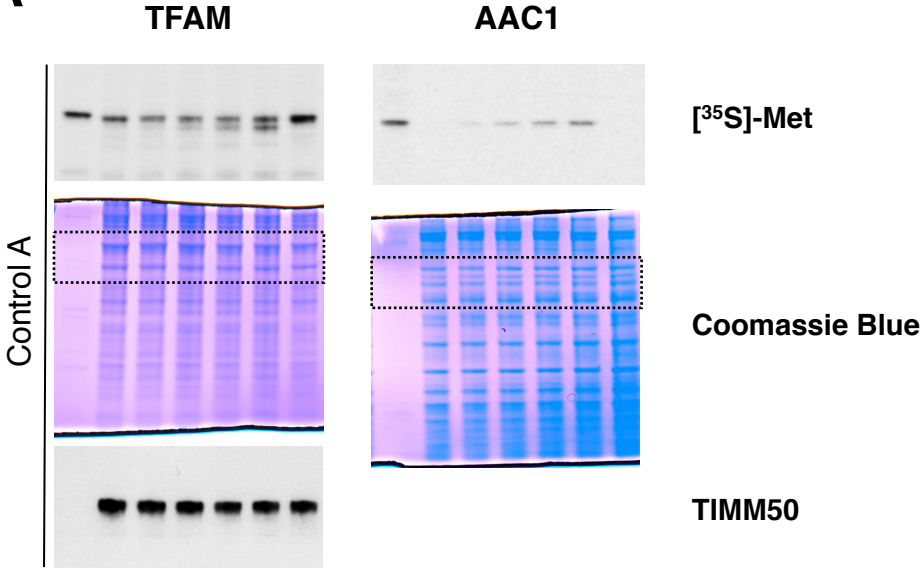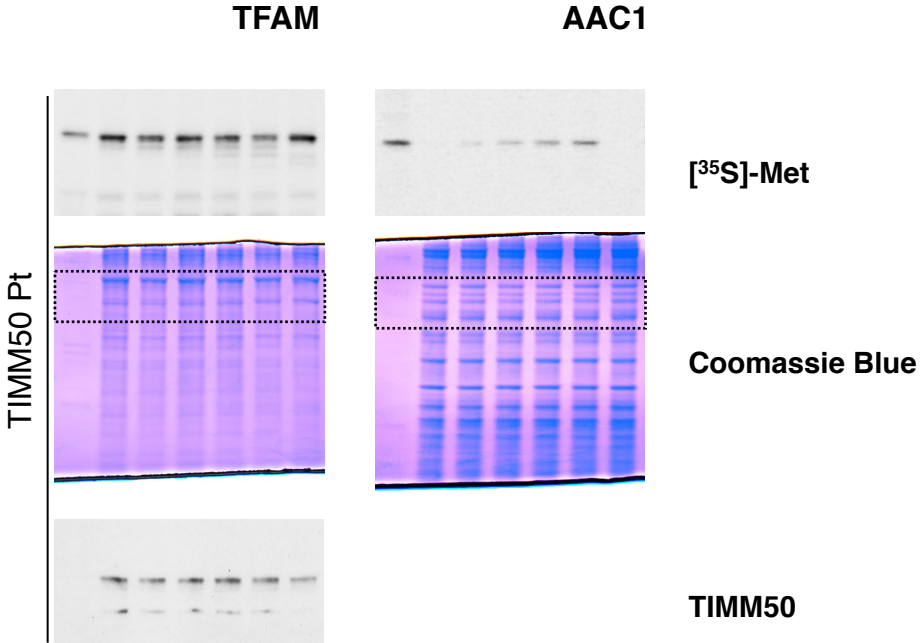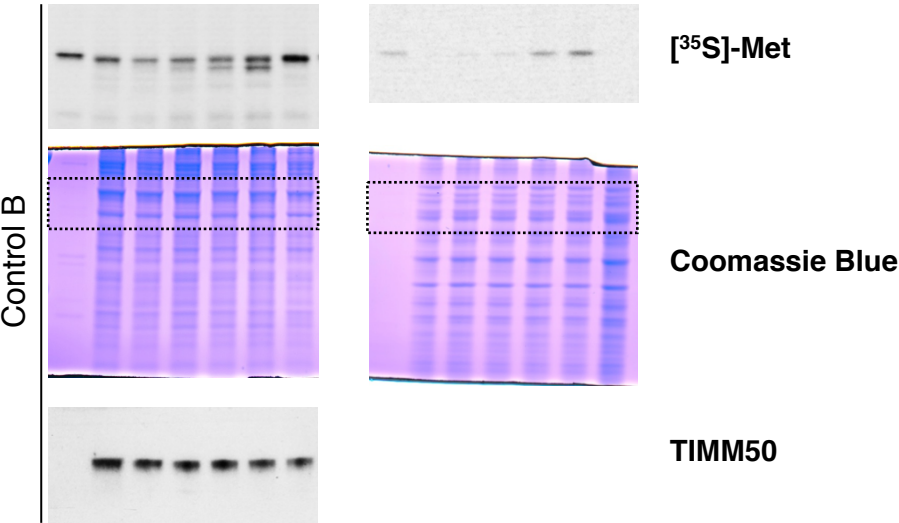

Supplement: Supplementary file 6 — Source Data for Figure 3 [file EMMM-10-e8698-s005.pdf]

4A

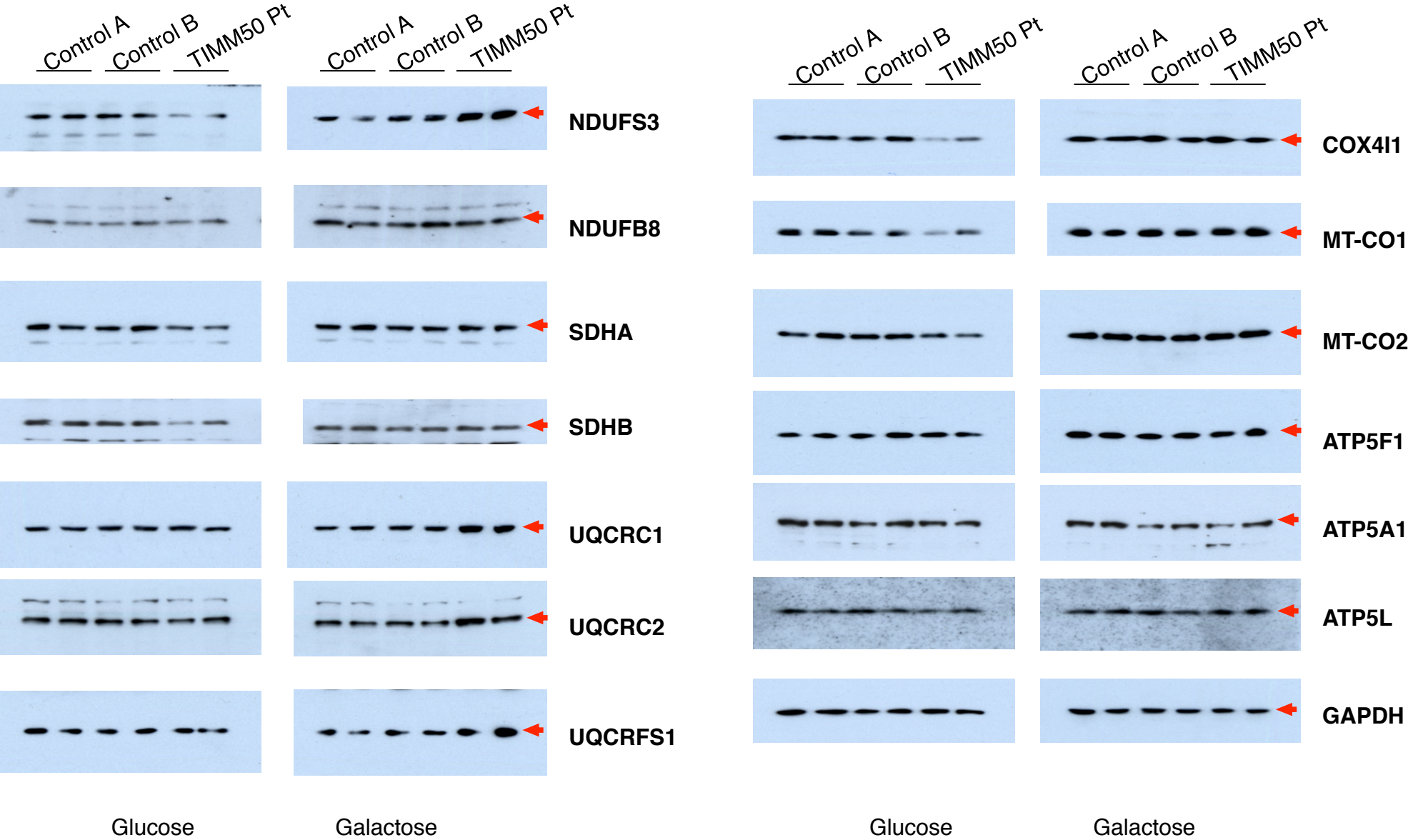

4B

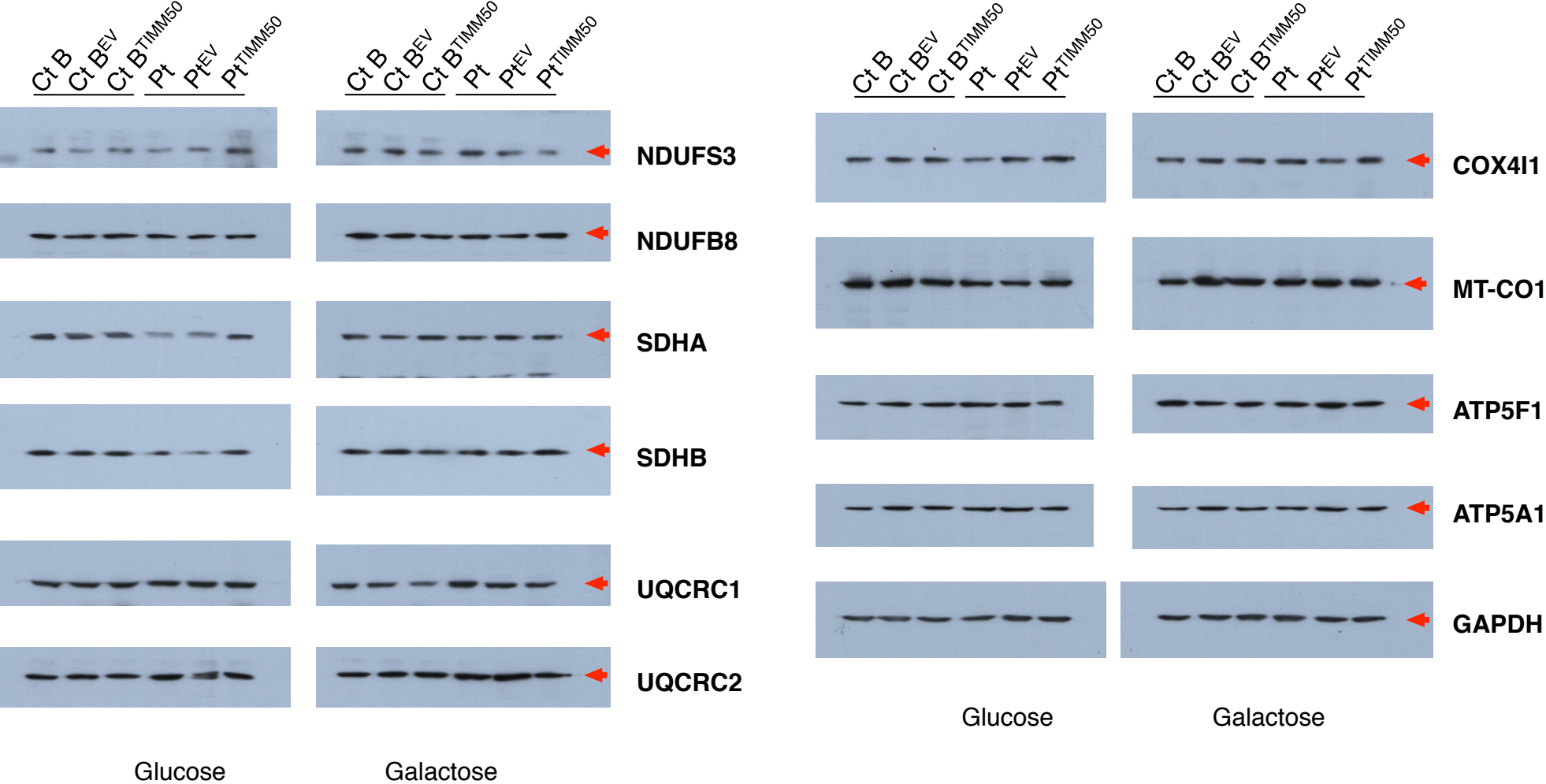

Supplement: Supplementary file 7 — Source Data for Figure 4 [file EMMM-10-e8698-s006.pdf]

5B

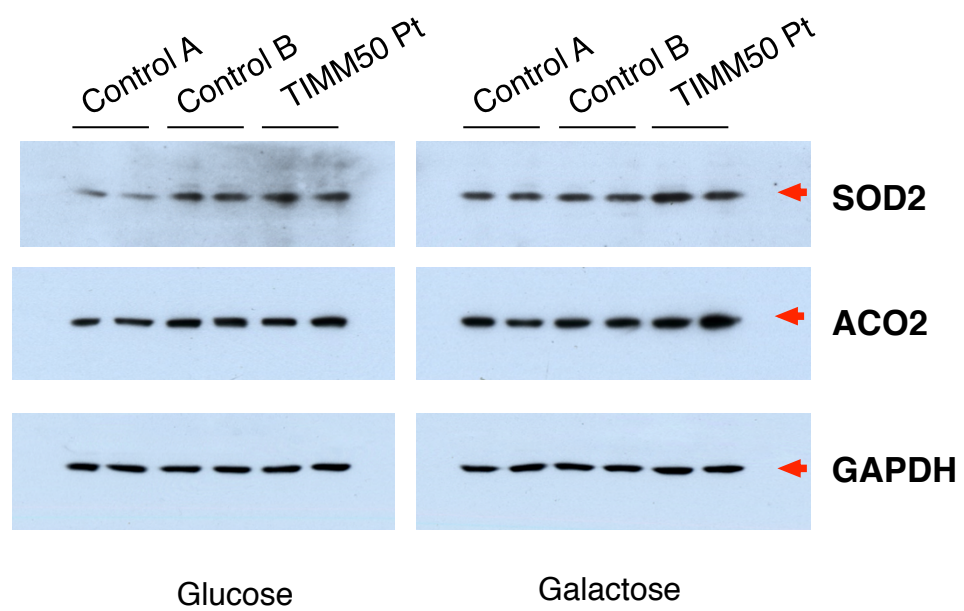

5D

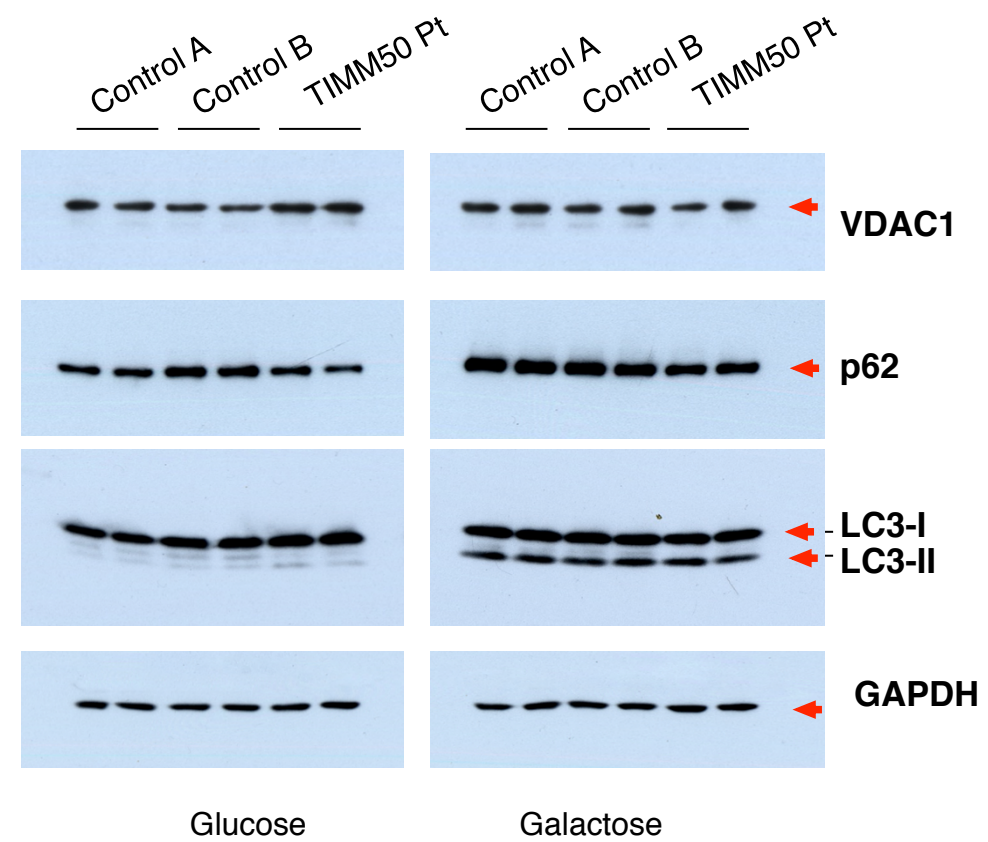

Supplement: Supplementary file 8 — Source Data for Figure 5 [file EMMM-10-e8698-s007.pdf]

7A

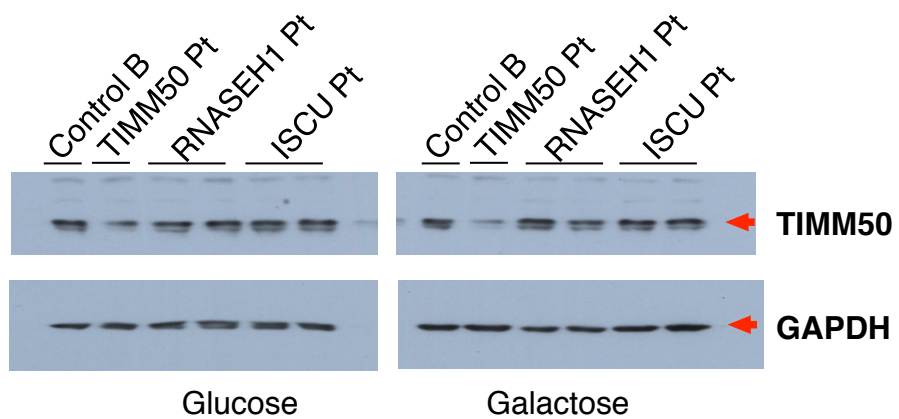

7E

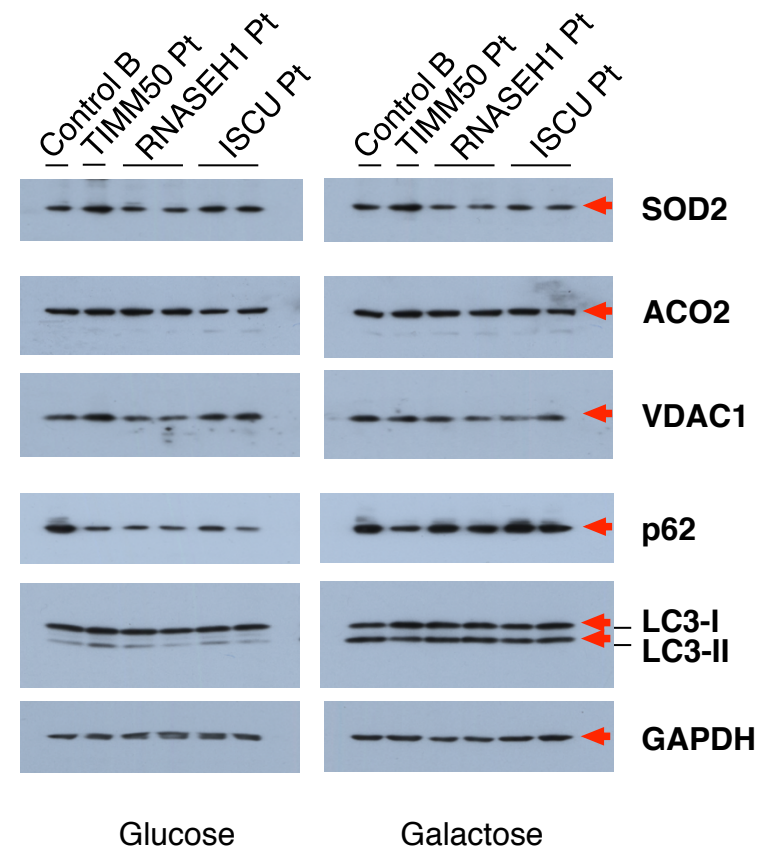

7C

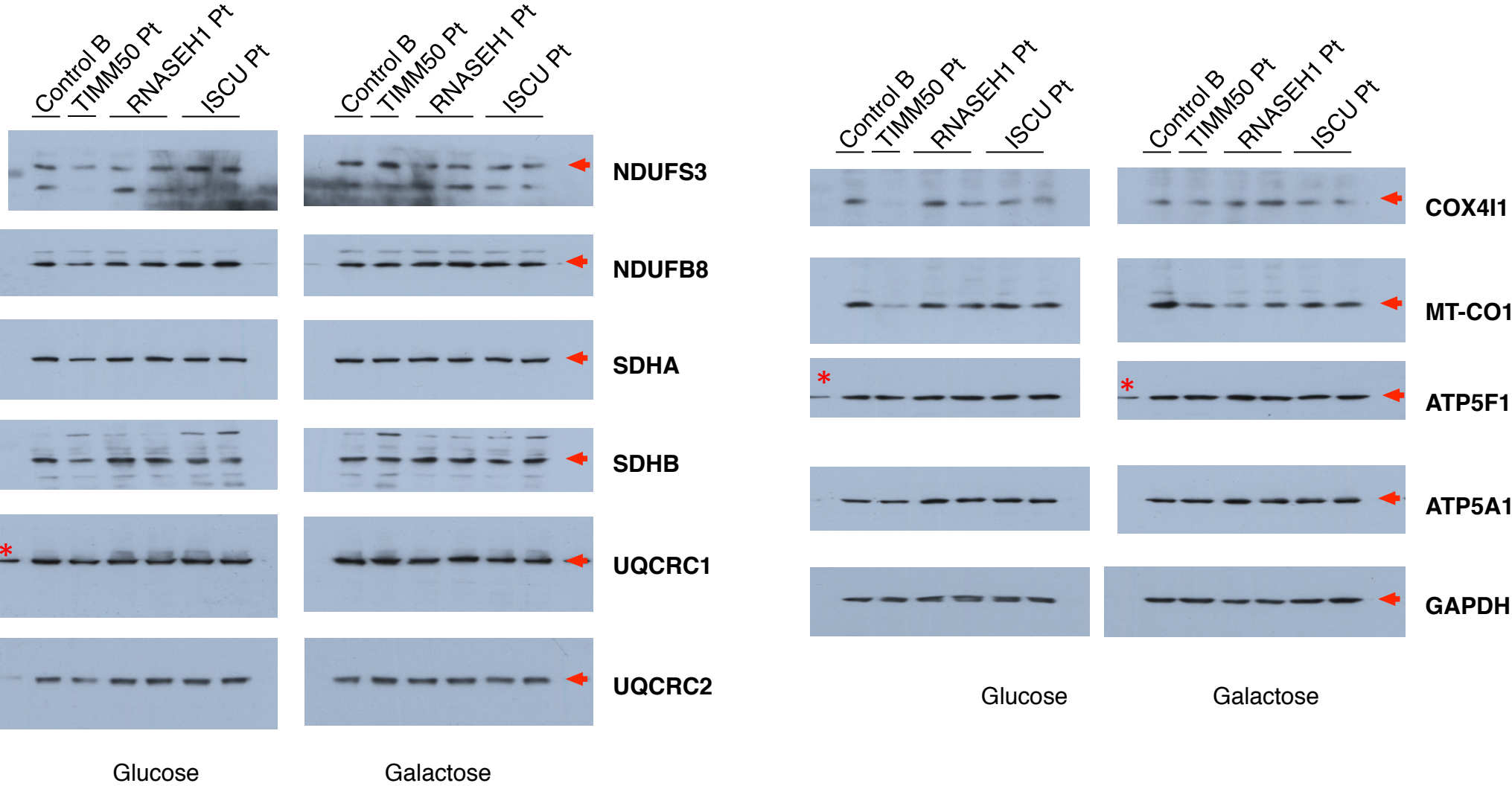

\* Unwanted lanes

Supplement: Supplementary file 9 — Source Data for Figure 7 [file EMMM-10-e8698-s008.pdf]
